# Cellular characterization of human epicardial adipose tissue: highly expressed PAPP‐A regulates insulin‐like growth factor I signaling in human cardiomyocytes

**DOI:** 10.14814/phy2.14006

**Published:** 2019-02-26

**Authors:** Cheryl A. Conover, Laurie K. Bale, Robert L. Frye, Hartzell V. Schaff

**Affiliations:** ^1^ Division of Endocrinology Mayo Clinic Rochester Minnesota; ^2^ Department of Cardiovascular Diseases Mayo Clinic Rochester Minnesota; ^3^ Department of Cardiovascular Surgery Mayo Clinic Rochester Minnesota

**Keywords:** Cardiomyocytes, epicardial fat, insulin‐like growth factors, pregnancy‐associated plasma protein‐A, subcutaneous fat

## Abstract

Little is known about the cellular biology of fat surrounding the human heart. In this study, we obtained paired samples of epicardial fat, the visceral fat depot attached to the heart, and subcutaneous skin fat from patients undergoing open heart surgery to test the hypothesis that human epicardial fat cells differentially express bioactive molecules that have the potential to affect cardiac function. First, we characterized the free fatty acids (FFAs), adipocytokines, and growth factors secreted by isolated adipocytes and preadipocytes in cell culture. There was little to distinguish the fat cell secretory products in terms of FFAs and adipocytokines. The most striking finding was that preadipocytes from epicardial adipose tissue expressed high levels of pregnancy‐associated plasma protein‐A (PAPP‐A), a novel metalloproteinase that enhances local insulin‐like growth factor (IGF) action through cleavage of inhibitory IGF binding protein‐4 (IGFBP‐4). PAPP‐A levels were 15‐fold higher in conditioned medium from epicardial preadipocytes than from subcutaneous preadipocytes (*P *<* *0.0001). PAPP‐A was not expressed in mature adipocytes. Next we determined whether PAPP‐A could affect IGF‐I signaling in a human cardiomyocyte cell line. IGF‐I activated receptor‐mediated auto‐phosphorylation, and this was blocked by wild‐type and protease‐resistant IGFBP‐4. Addition of PAPP‐A induced cleavage of wild‐type, but not protease‐resistant, IGFBP‐4 thereby restoring IGF‐I action. A proteolytically defective PAPP‐A had no effect. IGF‐I receptor‐mediated signaling through the phosphatidylinositol 3‐kinase pathway was similarly inhibited by IGFBP‐4 and restored by PAPP‐A. Thus, human epicardial fat cells differentially express PAPP‐A, which has the potential to affect IGF signaling in the heart.

## Introduction

Epicardial fat is the visceral fat depot of the heart in humans. It shares a common embryologic origin with other intra‐abdominal visceral fat depots. Epicardial fat directly contacts the myocardium with no intervening fascia, and shares the same microcirculation (Sacks and Fain 2007; Kaushik and Reddy 2011; Iacobellis 2015). It has been proposed that this intimate location of epicardial fat on the heart surface is integral to its function.

Epicardial adipose tissue is thought to have secretory functions similar to other visceral fat depots, and it has been hypothesized that its generation of bioactive molecules, such as free fatty acids (FFAs), cytokines, and growth factors, could affect cardiac function (Fitzgibbons and Czech 2014; Talman et al. 2014; Iacobellis 2015). Epicardial fat is easily detected and measured by imaging. There are several clinical studies showing that epicardial thickness is a proxy measure of visceral adiposity (Iacobellis et al. 2003a,b), and that it correlates with atherosclerosis, heart failure, atrial fibrillation, metabolic syndrome, insulin resistance, and fatty liver disease (Thanassoulis et al. 2010; Iacobellis et al. 2011, 2014; Fitzgibbons and Czech 2014; Iwayama et al. 2014; Talman et al. 2014; Iacobellis 2015). However, there are very few studies directly assessing human epicardial fat physiology and pathophysiology. Nevertheless, studies in mice indicate an important role for pregnancy‐associated plasma protein‐A (PAPP‐A) in regulating visceral fat depots (Conover et al. 2013; Bale et al. 2018). Although mice do not have epicardial fat per se, that is, fat in direct contact with the myocardium, they do have fat around the heart which has visceral characteristics and relatively high PAPP‐A expression (Bale et al. 2018).

PAPP‐A is a novel zinc metalloprotease that can increase local insulin‐like growth factor (IGF)‐I bioavailability through cleavage of inhibitory IGF binding proteins, in particular IGFBP‐4 (Conover 2012). PAPP‐A is a secreted protein that associates with the surface of cells through heparan sulfate‐like proteoglycans in an autocrine/paracrine fashion. IGF bound to IGFBP‐4 is unable to activate receptors. However, its binding to IGFBP‐4 induces a conformational change making IGFBP‐4 a substrate for PAPP‐A. Upon cleavage of IGFBP‐4 by PAPP‐A, IGF is liberated from the complex in the pericellular environment and IGF signaling is initiated. PAPP‐A‐induced enhancement of local IGF‐I action through proteolysis of IGFBP‐4 has been demonstrated in several in vitro and in vivo systems (Conover 2012). Increased IGF signaling has been associated with aging and many age‐related diseases (Bartke 2008). Conversely, inhibition of PAPP‐A expression or its proteolytic activity represents an innovative approach to decreasing IGF availability with moderate restraint of IGF‐I receptor signaling. We have shown that inhibition of PAPP‐A through gene deletion in mice has many beneficial effects, including a remarkable extension of lifespan by 30–40%, suppression of atherosclerotic plaque progression, and prevention of visceral obesity and fatty liver (Harrington et al. 2007; Conover et al. 2010, 2013). These effects occurred in both male and female PAPP‐A knock‐out mice. We can also inhibit the ability of PAPP‐A to cleave IGFBP‐4 in vitro and in vivo with a novel immunoneutralizing monoclonal antibody generated against a unique exosite in PAPP‐A (mAb‐PA 1/41) (Mikkelsen et al. 2008). Intraperitoneal injection of mAb‐PA 1/41 significantly inhibited atherosclerosis plaque progression in apolipoprotein E‐deficient mice by 70% (Conover et al. 2016).

Thus, PAPP‐A appears to be an important regulator of visceral fat and perhaps cardiovascular function. However, there have been no studies to date on PAPP‐A or IGF signaling and epicardial fat. It is of note that most in vitro studies of epicardial fat have used whole tissue explants (Mazurek et al. 2003; Karastergiou et al. 2010; Yerramasu et al. 2012), which would include adipocytes, preadipocytes, immune infiltrates, and endothelial cells. Importantly, preadipocytes from different regions of the body are inherently distinct, which likely contributes to differential fat distribution and function (Tchkonia et al. 2013). These cells retain their phenotypic and functional differences in short‐term culture. PAPP‐A is overexpressed in human, nonhuman primate, and mouse preadipocytes from visceral fat compared to subcutaneous fat (Tchkonia et al. 2007; Tchoukalova et al. 2009; Conover et al. 2013; Davidge‐Pitts et al. 2014; Gude et al. 2016). Adipocytes per se have seen limited investigation.

In this study we tested the hypothesis that human epicardial fat cells express bioactive molecules that affect cardiac function. For the first phase, we collected paired epicardial and subcutaneous adipose tissue samples from patients undergoing open heart surgery. Small sections of each sample were taken for lipid content. Adipocytes and preadipocytes were isolated from the remainder of the tissue sample for cell culture studies. This phase characterized human epicardial adipose tissue cellular composition and secretory products compared to subcutaneous adipose tissue. The second phase took the information derived from the first phase, that is, that a differentially expressed secretory product from epicardial fat cells was PAPP‐A, to assess PAPP‐A and IGF‐I effects on human cardiomyocytes in vitro.

## Materials and Methods

### Clinical samples

The following protocol was approved by the Institutional Review Board of Mayo Clinic (IRB# 16‐008336). Only patients on the surgical schedule of Hartzell V. Schaff were approached. CAC met with these patients, discussed the protocol, answered any questions, and, if the volunteers agreed, obtained written informed consent. Forty patients was the requested and approved accrual number and this number of patients was obtained between 14 June 2017 and 9 October 2017 at St. Marys Hospital of Mayo Clinic. Patients were de‐identified and only sex, age, and reason for surgery were collected. This information is presented in Table 1. On the day of the surgery, samples of epicardial fat (0.2–1.6 g), removed from the right atrial‐ventricular groove as part of the surgical procedure, and subcutaneous fat (0.4–3.3 g), removed from the chest incision site, were taken by HVS and placed in sterile transport medium for pick‐up from the operating room. Samples were processed immediately under aseptic conditions for preadipocyte and adipocyte cell culture and the different analyses. Only paired epicardial and subcutaneous samples were analyzed. Eleven of the paired patient samples were not assessed due to small sample size, few adipocytes, or failure of isolated preadipocytes to grow to sufficient numbers in culture.

**Table 1:** Study subjects

Male/female	14/15
Age	22–78 years
Surgeries	
Cardiomyopathy/septal myectomy	14
Valve replacement/repair	10
Coronary bypass	3
Other	2

Characteristics of patients undergoing open heart surgery who had given written informed consent to have epicardial and subcutaneous adipose tissue samples removed as part of the surgical procedure.

### Adipocyte size

Fresh samples of epicardial and subcutaneous fat were digested with collagenase in HEPES in a 37°C shaking water bath for 8–10 min or until fat globules were no longer present (Tchoukalova et al. 2003). Following centrifugation and methylene blue staining, size of the dispersed adipocytes was determined using a Nikon Labophot‐2, Melville, NY with Coolpix camera and software Analyze/Cell Counting and Application Processing. Circularity, diameter, and area measurements were computed. Images were analyzed until ~300 cells were sized.

### Materials for cultures

Fetal bovine serum (FBS), horse serum (HS), bovine serum albumin (BSA), AC16 human cardiomyocyte cell line (Sigma, St. Louis, MO); *α*MEM, DMEM/F12, penicillin, streptomycin, gentamycin, amphotericin, l‐glutamine, HBSS, HEPES, erythrocyte lysing buffer, phosphorylated IGF‐I receptor antibody (Thermo Fisher Scientific/Gibco, Waltham, MA); Total IGF‐I receptor antibody (Novo Biologicals, Littleton, CO); Collagenase type II (Worthington Biochemical Corporation, Lakewood, NJ); IGF‐I, IGF‐II (R&D Systems, Minneapolis, MN); IGFBP‐4 antibodies, phosphorylated Akt antibody, total, and phosphorylated ERK1/2 antibody (Abcam, Cambridge, MA); Total Akt antibody (Cell Signaling, Danvers, MA); Fluorescently labeled secondary antibodies (LI‐COR, Lincoln, NE); IgG2a (Bio X Cell, West Lebanon, NH). Ultrasensitive human PAPPA ELISA (picoPAPP‐A; AL‐101), total human IGFBP‐4 ELISA (AL‐126), and human IGF‐I (AL‐121) ELISA kits as well as an inhibitory PAPPA monoclonal antibody (mAb‐PA 1/41) were generous gifts of Ansh Laboratories (Webster, TX). Recombinant human IGFBP‐4 (wild‐type and protease‐resistant) and recombinant human PAPP‐A (wild‐type and mutated for markedly reduced proteolytic activity) were kindly provided by Professor Claus Oxvig (Aarhus University, Denmark).

### Adipocyte and preadipocyte cell culture

Human adipose tissue samples (epicardial and subcutaneous) collected in the operating room were put into preweighed sterile tubes containing transport media (HBSS with gentamycin, amphotericin, penicillin, streptomycin and l‐glutamine, pH 7.4). Tubes were weighed again to determine the weight of the tissue, which was removed to another tube containing collagenase (3 mg/g tissue) in HBSS with 3.5% BSA and minced to a fine consistency. Samples were then incubated at 37°C in a shaking water bath for 30–40 min with vortexing every 5 min, filtered through 100 *μ*m nylon mesh and centrifuged 349 *g* for 10 min at room temperature. Adipocytes floating on the top were collected using a wide‐bore pipet tip. Remaining supernatant was gently aspirated, and the pellet resuspended with 5–10 mL erythrocyte lysing buffer and incubated in a shaking water bath at 37°C for 5 min. This was then centrifuged at 242 *g* for 10 min, the supernatant aspirated, and the pellet resuspended in *α*MEM containing 10% FBS, glutamine, penicillin, and streptomycin for plating. Preadipocytes at passage 3–7 were used for experiments. At confluency, the preadipocytes were washed twice and changed to *α*MEM without serum for 48 h. The adipocytes from the same patient were incubated in the serum‐containing medium for 2 h and then changed to the serum‐free medium for 48 h. Preadipocyte cell number and adipocyte DNA were used for normalizing results of the conditioned media analyses.

### Lipolysis

Lipolysis was measured by analyzing 48‐h serum‐free conditioned medium from epicardial and subcutaneous adipocytes for free glycerol content using a commercial kit (High Sensitivity Free Glycerol Assay Kit, Sigma‐Aldrich, St. Louis, MO). In addition, FFAs of various lengths and saturation were measured in the Mayo Clinic Metabolomics Core using triple quadrupole mass spectrometers and authentic isotopically labeled standards for quantitation.

### Adipocytokines

Preadipocyte and adipocyte conditioned media were analyzed using a MILLIPLEX Human Adipocyte Magnetic Bead Panel (MilliporeSigma, Burlington, MA) by the Immunochemical Core Laboratory of Mayo Clinic.

### PAPP‐A

Ultrasensitive ELISA kits that measure human PAPP‐A were used to determine levels of PAPP‐A in 48‐h conditioned media. For proteolysis assays, human IGFBP‐4 (4 nmol/L) precomplexed to IGF‐II (2 nmol/L) was incubated in cell‐free conditioned medium without and with inhibitory PAPP‐A monoclonal antibody (mAb‐PA 1/41, 10^−7^ mol/L) for 2 h at 37°C, as previously described (Mikkelsen et al. 2014). IGF must be bound to IGFBP‐4 for IGFBP‐4 to be a substrate for PAPP‐A proteolysis, and IGF‐II is more effective than IGF‐I in this cell‐free assay (Conover et al. 1993; Qin et al. 2000; Laursen et al. 2001). mAb‐PA 1/41 specifically inhibits PAPP‐A‐mediated IGFBP‐4 proteolysis (Mikkelsen et al. 2014). Western blotting was performed as previously described (Mikkelsen et al. 2014). Briefly, samples were separated by 12% SDS‐PAGE under reducing conditions, blotted onto a PVDF membrane, blocked, and probed for IGFBP‐4 fragments using specific N‐ and C‐terminal antibodies. Fluorescently labeled secondary antibodies were used for detection of intact and cleaved IGFBP‐4 by LI‐COR Odessey.

### Cardiomyocytes

The AC16 human cardiomyocyte cell line was derived from fusion of primary cells from adult ventricular heart tissue with SV40‐transformed human fibroblasts. AC16 human cardiomyocytes proliferate when cultured in DMEM/F12 with 12.5% FBS. They differentiate when cultured for 5 days in mitogen‐free medium (DMEM/F12 with 2% HS) on plates coated with nitrocellulose. Cells were then washed and changed to serum‐free medium (DMEM/F12 with 0.1% BSA) for 48 h before experiments.

### IGF‐I receptor phosphorylation

IGF‐I (2 nmol/L) was preincubated at 37°C for 6 h in serum‐free medium alone or in combinations with IGFBP‐4 (4 nmol/L) and PAPP‐A (60 ng/mL) before adding the incubated media to the AC16 cardiomyocytes. After a 10‐min stimulation with preincubated IGF‐I, cardiomyocytes were lysed in 125 *μ*L RIPA buffer (0.15 M NaCl, 0.5% NP‐40, 0.1% SDS, 50 mM Tris) containing phosphatase and protease inhibitors. Sample proteins were separated by 7.5% SDS‐PAGE under reducing conditions, transferred to PVDF, blocked in 3% BSA/TBS‐T, and incubated overnight at 4°C with primary antibody (against phosphorylated or total *β*‐subunit of the IGF‐I receptor). Membranes were then washed, incubated with secondary antibody (HRP‐conjugated goat anti‐rabbit; Jackson Immunoresearch Laboratories, West Grove, PA), and imaged using enhanced chemiluminescence (GE Healthcare, Piscataway, NJ). Details can be found in Donegan et al. (2016).

### IGF‐I receptor‐mediated signaling

Cell‐free preincubation with IGF‐I, IGFBP‐4, and PAPP‐A for 6 h was as described under IGF‐I receptor phosphorylation. Cells were washed and stimulated with the preincubated medium for 10 min. Cells were lysed and processed as for IGF‐I receptor phosphorylation, except that PVDF membranes were probed with antibodies to total and phosphorylated Akt and ERK1/2. LI‐COR software was used to quantitate bands in western blots.

### Statistical analyses

Results are expressed as mean ± SEM. We used paired *t*‐test to compare epicardial and subcutaneous samples from the same patient. If there was not enough of one sample for an assay, the pair was not included. We used ANOVA followed by Dunnett's for quantitative western blots. Significance was set at *P *<* *0.05.

## Results

### Study subjects

Table 1 presents the sex, age, and surgery distribution of the study subjects. There were 14 males and 15 females included in the study (11 subjects had to be omitted for technical reasons with one of the paired cell cultures, as discussed in Material and Methods. The age range was 22–78 years. The majority of the surgeries (>80%) were for hypertrophic cardiomyopathies and valve replacement/repair.

### Whole‐adipose tissue analyses

The lipid content of adipocytes from epicardial adipose tissue was ~50% less than those from subcutaneous adipose tissue (*P *<* *0.0001; Table 2). This did not differ between sexes. Lipid content tended to be less in the younger aged subjects, but this was significant only for epicardial adipose tissue (*P *=* *0.005).

**Table 2:** Lipid content of adipocytes

	Lipid (*μ*g per cell)		
	Epicardial	Subcutaneous	*P*‐value
Overall	0.33 ± 0.021	0.67 ± 0.049	<0.0001
Male/female	0.38 ± 0.032/0.34 ± 0.028	0.63 ± 0.068/0.64 ± 0.064	NS/NS
Age (20–50/51–80)	0.26 ± 0.017/0.037 ± 0.030	0.57 ± 0.067/0.69 ± 0.061	0.005/NS

Adipocytes were isolated from patient epicardial and subcutaneous fat, stained, and analyzed as described in Materials and Methods. Results are means ± SEM, *N* = 28. Data were analyzed by paired *t*‐test. NS, not significant.

### Adipocyte and preadipocyte cell culture

#### Fatty acids

The FFA profile of 48‐h conditioned medium from epicardial and subcutaneous adipocytes is summarized in Table 3. The only significant difference was in linolenic acid, which comprised only 0.25% of the total FFAs; its concentration was lower in epicardial conditioned medium compared to subcutaneous medium. The major FFAs, linoleic, palmitic, oleic, and steric acids, comprised ~90% of the total FFAs in both epicardial and subcutaneous adipocyte conditioned medium. Using free glycerol in the medium as a measure of lipolysis, epicardial‐adipocyte‐conditioned medium appeared to have twice as much free glycerol than subcutaneous (16,278 ± 6228 and 7211 ± 3563 pmol/mL, respectively), but because of the variability this did not result in a statistically significant difference. Although turnover appeared to be greater in epicardial versus subcutaneous adipocytes, the profile of FFAs did not differ substantially between the adipocytes from two different types of fat depots.

**Table 3:** FFAs in adipocyte conditioned medium

FFA	% of total	
	Epicardial	Subcutaneous
EPA	0.37	0.25
Linolenic	0.25 (*P *=* *0.017)	3.17
DHA	1.12	0.56
Myristic	4.91	3.65
Palmitoleic	3.24	3.71
Arachidonic	1.79	1.43
Linoleic	21.57	23.64
Palmitic	16.62	17.08
Oleic	31.57	36.30
Steric	18.49	10.22

Forty‐eight hours serum‐free conditioned media from adipocytes isolated from patient epicardial and subcutaneous fat. *N* = 12. FFA, free fatty acid; EPA, eichosapentaenoic acid; DHA, docosahexaenoic acid.

#### Adipocytokines

We used a multiplex system to measure cytokines and other factors in 48‐h conditioned medium from epicardial and subcutaneous preadipocytes and adipocytes. We were looking for differences between epicardial and subcutaneous secretory products. In conditioned medium from adipocytes (Table 4), there were high concentrations of interleukin (IL)‐6, leptin, adiponectin, and plasminogen inhibitor (PAI)‐1, but no significant differences in these adipocytokines between epicardial and subcutaneous cells. IL‐8 and monocyte chemoattractant protein (MCP‐1) were part of the human adipocyte panel, but readings were above the limits of quantitation in this multiplex assay and, therefore, not included. Tumor necrosis factor (TNF)‐*α*, IL‐1*β*, nerve growth factor (NGF), hepatocyte growth factor (HGF), and resistin were below the limits of quantitation using this system.

**Table 4:** Cytokines/Adipokines in Adipocyte Conditioned Media

	pg/mL per ng DNA		*P*‐value
	Epicardial	Subcutaneous	
NGF	BLD	BLD	
IL‐6	20,302 ± 6946	12,721 ± 5564	0.728
Leptin	11,446 ± 3466	11,204 ± 3969	0.568
HGF	BLD	BLD	
Adiponectin	20,302 ± 6946	11,446 ± 3466	0.501
TNF*α*	BLD	BLD	
Resistin	BLD	BLD	
IL‐1*β*	BLD	BLD	
PAI‐1	10,837 ± 2280	5733 ± 2435	0.058

Forty‐eight hours serum‐free conditioned media from adipocytes isolates from patient epicardial and subcutaneous fat. Results are mean ± SEM, *N* = 9. Data were analyzed by paired *t*‐test. BLD, below the limits of quantitation in the MILLIPLEX Human Adipocyte Magnetic Bead Panel; NGF, nerve growth factor; HGF, hepatocyte growth factor; TNF, tumor necrosis factor; IL, interleukin; PAI, plasminogen inhibitor.

In conditioned medium from preadipocytes (Table 5), IL‐6 and HGF concentrations were significantly greater in epicardial cell conditioned medium than in subcutaneous conditioned medium. Levels of PAI‐1 were high but not significantly different in conditioned medium between epicardial and subcutaneous preadipocytes. As with the adipocyte conditioned media, IL‐8 and MCP‐1 readings were above the limits of quantitation in preadipocyte conditioned medium and not included. Other analytes on this panel, that is, leptin, adiponectin, TNF‐*α*, IL‐1*β*, NGF, and resistin were below the level of detection.

**Table 5:** Cytokines/Adipokines in Preadipocyte Conditioned Media

	pg/mL per 10^5^ cells		*P*‐value
	Epicardial	Subcuatanous	
NGF	BLD	BLD	
IL‐6	13,607 ± 1297	8720 ± 2030	0.0.32
Leptin	BLD	BLD	
HGF	506 ± 138	77 ± 11	0.021
Adiponectin	BLD	BLD	
TNF*α*	BLD	BLD	
Resistin	BLD	BLD	
IL‐1*β*	BLD	BLD	
PAI‐1	32,063 ± 5295	16,335 ± 1825	0.060

Forty‐eight hours serum‐free conditioned media from preadipocytes isolates from patient epicardial and subcutaneous fat. Results are mean ± SEM, *N* = 9. Data were analyzed by paired *t*‐test. BLD: below the limits of quantitation in the MILLIPLEX Human Adipocyte Magnetic Bead Panel; NGF, nerve growth factor; HGF, hepatocyte growth factor; TNF, tumor necrosis factor; IL, interleukin; PAI, plasminogen inhibitor.

#### PAPP‐A

**Figure 1:**
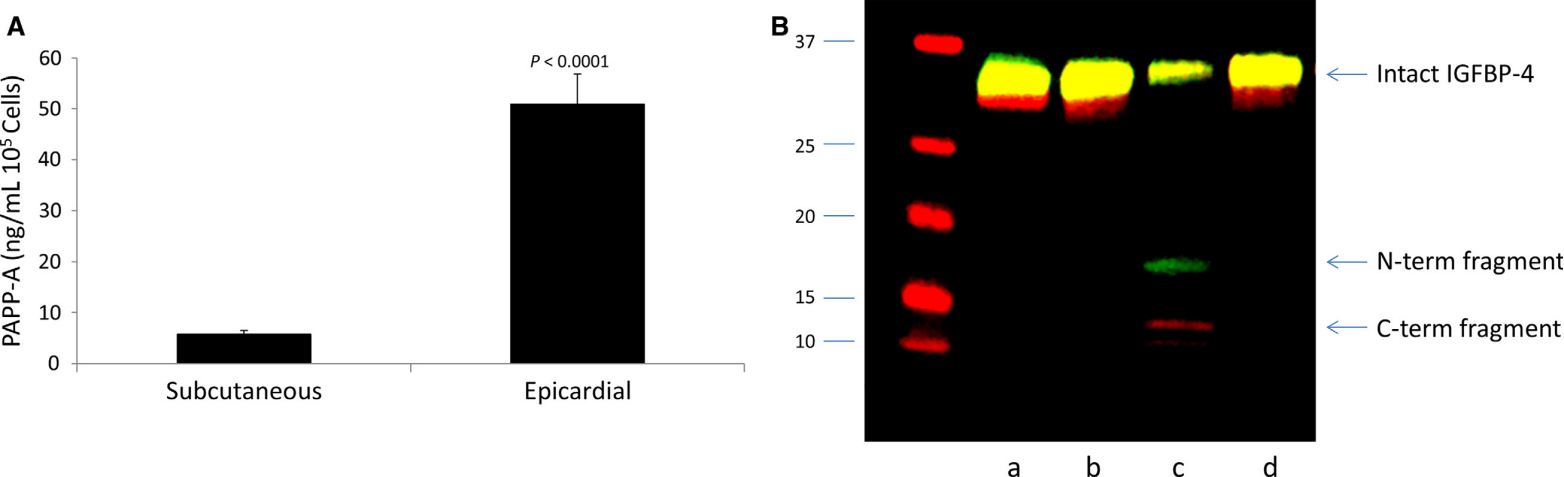
(A) PAPP‐A protein levels in conditioned medium from subcutaneous and epicardial preadipocytes. Cells were washed and changed to serum‐free medium for 48 h. PAPP‐A levels were measured by ELISA. Results are mean ± SEM,* N* = 29. Data were analyzed by paired *t*‐test. (B) PAPP‐A‐mediated IGF‐dependent IGFBP‐4 proteolysis in epicardial preadipocyte CM. IGFBP‐4 is included in all lanes. (a) *t* = 0; (b) CM; (c) CM + IGF‐II; (d) CM + IGF‐II + mAb‐PA 1/41. Arrows indicate Intact and the N‐ and C‐terminal fragments of IGFBP‐4. Molecular weight markers are indicated on the left. PAPP‐A, pregnancy‐associated plasma protein‐A; IGF, insulin‐like growth factor; IGFBP‐4, IGF binding protein‐4; CM, conditioned medium.

Since PAPP‐A has been shown to be overexpressed in visceral fat compared to subcutaneous fat (Conover et al. 2013; Tchkonia et al. 2013; Davidge‐Pitts et al. 2014; Gude et al. 2016; Bale et al. 2018), we measured PAPP‐A in medium conditioned by epicardial and subcutaneous fat cells. Adipocytes from epicardial and subcutaneous fat did not express PAPP‐A. However, in paired analyses PAPP‐A levels in conditioned medium from epicardial preadipocytes were a remarkable 15‐fold higher (*P *<* *0.0001) than those in subcutaneous preadipocytes. Mean levels were 50.9 ± 5.97 and 5.8 ± 0.73 ng/mL per 10^5^ cells, respectively (Fig. 1A). This PAPP‐A was proteolytically active against IGFBP‐4 in an IGF‐dependent manner, and could be inhibited with mAb‐PA 1/41, a specific immunoneutralizing monoclonal antibody against PAPP‐A (Mikkelsen et al. 2014). A representative Western blot indicating intact IGFBP‐4 and the N‐ and C‐terminal IGFBP‐4 fragments generated by PAPP‐A‐mediated proteolysis is presented in Figure 1B. This result was reproduced in epicardial conditioned medium from four subjects. Thus, we focused on PAPP‐A‐modulated IGF signaling and its potential influence on heart function.

### Cardiomyocyte cell culture

**Figure 2:**
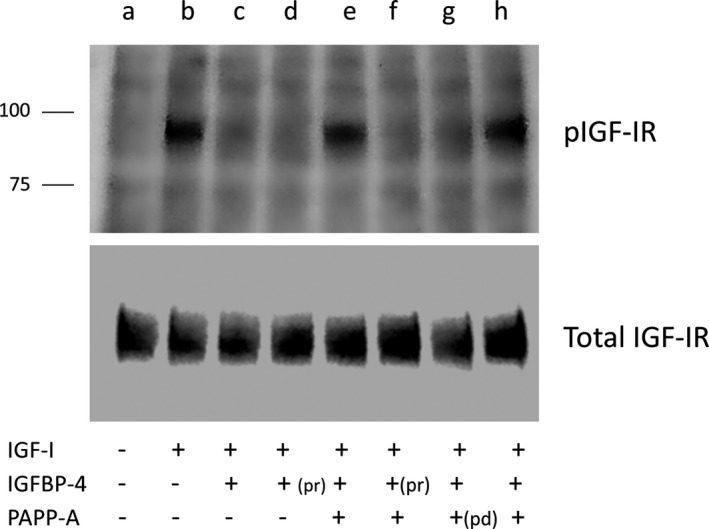
IGF‐I receptor activation in human cardiomyocytes: effect of IGF‐I and modulation by IGFBP‐4, PAPP‐A. Upper panel: Phosphorylated IGF‐I receptor 95 kDa *β*‐subunit. Lower panel: Total IGF‐I receptor. pr: protease‐resistant IGFBP‐4. pd: proteolytically defective PAPP‐A. Molecular weight markers are indicated on the left. IGF, insulin‐like growth factor; IGFBP‐4, IGF binding protein‐4; PAPP‐A, pregnancy‐associated plasma protein‐A.

AC16 human cardiomyocytes were cultured in differentiation medium for 5 days, and then changed to serum‐free medium for 48 h, washed and incubated with 0, 1, 2, 5, 10 nmol/L IGF‐I for 10 min. Western blot of dose‐dependent IGF‐I stimulation of IGF‐I receptor activation, that is, autophosphorylation of tyrosines on the 95 kDa *β*‐subunit, indicated that a maximal effect was achieved between 2 and 5 nmol/L IGF‐I. IGF‐I had no effect on total IGF‐I receptor protein (data not shown). To determine the effect of PAPP‐A on IGF‐I receptor activation in cardiomyocytes, cells were stimulated with media that had been preincubated without additions or with IGF‐I; IGF‐I and wild‐type IGFBP‐4; IGF‐I and protease‐resistant IGFBP‐4; IGF‐I, wild‐type IGFBP‐4, and PAPP‐A; IGF‐I, protease‐resistant IGFBP‐4, and PAPP‐A; IGF‐I, wild‐type IGFBP‐4 and proteolytically defective PAPP‐A (see Materials and Methods for concentrations). IGF‐I alone produced a marked increase, ~20‐fold, in IGF‐I receptor phosphorylation (Fig. 2, lane b vs. a). This was completely inhibited if IGF‐I was preincubated with wild‐type or protease‐resistant IGFBP‐4 without added PAPP‐A (Fig. 2, lanes c and d). This finding indicated that the cardiomyocytes themselves express little or no PAPP‐A, which was confirmed in PAPP‐A ELISA (data not shown). Without PAPP‐A during the preincubation, IGF‐I would bind and remain bound to IGFBP‐4 and, in this state, unavailable for receptor activation. However, when active PAPP‐A was included in the preincubation with IGF‐I and wild‐type IGFBP‐4 (Fig. 2, lanes e and h), IGFBP‐4 cleavage was induced and IGF‐I was available for receptor binding and activation when the preincubation medium was added to the cells. If protease‐resistant IGFBP‐4 was present, it was not cleaved thereby inhibiting IGF‐I action (Fig. 2, lane f). Also, proteolytically defective PAPP‐A did not increase IGF‐I bioavailability in the presence of wild‐type IGFBP‐4 (Fig. 2, lane g). There was no change in total IGF‐I receptor protein in cardiomyocytes with any of the stimulations. Therefore, AC16 human cardiomyocytes are responsive to IGF‐I and their responsivity is acutely modulated by IGFBP‐4 and PAPP‐A.

**Figure 3:**
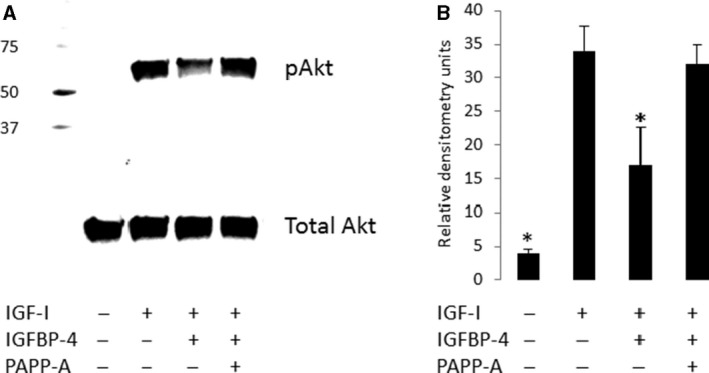
IGF‐I‐stimulated intracellular signaling in human cardiomyocytes: modulation by IGFBP‐4, PAPP‐A. (A) Upper panel: Phosphorylated Akt. Lower panel: Total Akt. Molecular weight markers are indicated on the left. (B) Quantitation of western blots, *N* = 3 separate experiments. *P < 0.05 compared to IGF‐I alone. IGF, insulin‐like growth factor; IGFBP‐4, IGF binding protein‐4; PAPP‐A, pregnancy‐associated plasma protein‐A.

We further explored IGF‐I receptor‐activated intracellular signaling in human cardiomyocytes, focusing on phosphatidylinositol 3‐kinase (PI3‐K) and mitogen‐activated protein kinase (MAPK) pathways. Phosphorylation of 60 kDa Akt at serine 473 and 44/42 kDa ERK1/2 at threonine 185 were used as read‐outs for PI3‐K and MAPK signaling pathways, respectively. There was little phosphorylated Akt under basal conditions, but in a dose–response, time course experiment 2 nmol/L IGF‐I stimulated a 10‐fold increase within 10 min and this increase was sustained over a 60 min period (data not shown). There was no change in total Akt with IGF stimulation. There was little if any IGF‐I stimulation of ERK1/2 phosphorylation (data not shown). As with IGF‐I receptor phosphorylation, IGF‐I stimulation of Akt phosphorylation was significantly inhibited ~50% by IGFBP‐4 (*P *=* *0.04), but this phosphorylation was restored when PAPP‐A was present (Fig. 3).

## Discussion

The two major findings of this study were that (1) the zinc metalloproteinase, PAPP‐A, is highly expressed and secreted by preadipocytes from human epicardial fat, and (2) PAPP‐A's proteolytic activity can regulate IGF‐I signaling in human cardiomyocytes. The latter is noteworthy in that IGF‐I signaling has been shown to be a key player in cardiac physiology and pathophysiology (Saetrum Opgaard and Wang 2005; Reddy et al. 2007; Troncoso et al. 2014). Bioactive products from epicardial fat have been hypothesized to interact locally with the myocardium through paracrine or vasocrine secretions (Iacobellis and Bianco 2011; Talman et al. 2014; Iacobellis 2015). Our data suggest that PAPP‐A could be such a locally acting factor.

We made these discoveries through the basic characterization of paired epicardial and subcutaneous fat cells (adipocytes and preadipocytes) from unselected patients undergoing elective open heart surgery. The majority of surgeries were for hypertrophic cardiomyopathies and valve replacement or repair. None of the patients were morbidly obese and only a few were undergoing surgery for cardiovascular disease. Indeed, several of the patients, especially the younger ones, appeared lean and physically fit, which was reflected in their lower epicardial adipocyte lipid content. An autopsy study also showed less epicardial adipose tissue in younger subjects (Iacobellis and Bianco 2011). Interestingly, there was no sex difference in lipid content in epicardial or subcutaneous adipocytes. Although the patients varied in sex, age, and surgical procedure, as well as in other aspects for which information was not collected (e.g., weight, ethnicity, medications), we compared data from the epicardial cells to the subcutaneous cells from the same patient. This experimental design allowed for paired analyses. We were looking for differences between epicardial and subcutaneous fat that might provide clues to a distinctive biology of epicardial fat.

We first looked at FFAs, the favored fuel for contractile function of heart muscle (Fitzgibbons and Czech 2014), and found that there were no major differences in the type or abundance of FFAs in the medium conditioned by epicardial and subcutaneous adipocytes. We presented these data to be complete in our characterization of the fat tissues, but did not pursue this aspect further.

We also used a commercial multiplex format to measure distinct analytes in the conditioned medium from both adipocytes and preadipocytes. In general, there was little apparent difference in basal secretion in the epicardial and subcutaneous adipocytes. However, epicardial preadipocytes expressed significantly more HGF than subcutaneous preadipocytes (adipocytes did not express HGF). Interestingly, HGF has been shown to have migratory and proangiogenic properties and, in conjunction with the proliferative effects of IGF‐I, HGF can promote myocardial repair in several animal models (Urbanek et al. 2005; Rota et al. 2008; Ellison et al. 2011). This warrants further study. IL‐6 secretion was also significantly greater in epicardial versus subcutaneous preadipocytes. However, adipocytes from both depots expressed very high levels of IL‐6, which would be expected to mask any differential effects in vivo. We were somewhat surprised not to find TNF*α* or IL‐1*β* in adipocyte or preadipocyte culture media, since several studies have reported high expression of these proinflammatory cytokines in epicardial fat (Mazurek et al. 2003; Ouwens et al. 2010; Yerramasu et al. 2012; Talman et al. 2014). It is of note that the data from those studies were derived from tissue explants, suggesting contribution of other cells in the adipose tissue such as inflammatory infiltrates. Indeed, activated macrophages are known to express high levels of TNF*α* and IL‐1*β*, and these proinflammatory cytokines are potent stimulators of PAPP‐A expression in human preadipocytes (Davidge‐Pitts et al. 2014).

Despite age, sex, and procedural differences there was remarkable consistency in that in all 29 subjects, PAPP‐A levels in the conditioned medium of epicardial preadipocytes greatly exceeded those of subcutaneous preadipocytes, resulting in a highly significant 15‐fold difference by paired *t*‐test (*P *<* *0.0001). This differential PAPP‐A expression appears to be inherent to the cells, since they were cultured and passaged in the same defined serum‐free medium. It will be of interest to investigate the regulation of PAPP‐A expression in these isolated preadipocytes.

From the discovery results, we chose to focus on PAPP‐A and its potential effect on IGF‐I signaling in human cardiomyocytes. To our knowledge, there are no reports describing direct effects of secretory products from human epicardial fat on cardiac function. The A16 cardiomyocytes do not produce detectable PAPP‐A, IGF‐I, or IGFBP‐4 under basal conditions, as measured by specific ELISAs (data not shown). Therefore, we were able to use this human cardiomyocyte model to simulate what might happen when PAPP‐A, IGF‐I, and IGFBP‐4 are made available from other cells in a paracrine manner, for example, epicardial preadipocytes, cardiac fibroblasts, and cardiac progenitor cells (Swifka et al. 2008; D'Elia et al. 2013; Barile et al. 2018). We found that IGF‐I was a potent activator of IGF‐I receptor signaling. This activity could be blocked by IGFBP‐4, but restored in the presence of active PAPP‐A via proteolysis of IGFBP‐4. Binding of IGF‐I to the extracellular *α*‐subunit of the IGF‐I receptor triggers autophosphorylation of the intracellular *β*‐subunit initiating intracellular signaling cascades (Girnita et al. 2014; Hakuno and Takahashi 2018). The two best characterized are PI3‐K/Akt and MAPK/ERK1/2 pathways. There was a robust Akt phosphorylation response to IGF‐I that could be modulated by IGFBP‐4 and PAPP‐A. There was little in the way of ERK1/2 phosphorylation with IGF‐I treatment. PI3‐K/Akt signaling can affect various bioactivities such as cell growth, survival, migration, and metabolism (Girnita et al. 2014; Hakuno and Takahashi 2018).

This study clearly shows that PAPP‐A can enhance IGF‐I signaling in cardiomyocytes. However, these in vitro experiments are unable to tell us whether elevated PAPP‐A and IGF signaling is “good” for the heart or “bad” for it. There are data to support both sides of the argument. There are several animal studies showing that IGFs are important in cardiac repair (Reddy et al. 2007; Rota et al. 2008). A recent paper suggested that cardioprotection by cardiac progenitor cell‐secreted exosomes was dependent on active PAPP‐A on the exosome surface. PAPP‐A‐mediated IGF‐I release via proteolytic cleavage of IGFBP‐4 contributed to angiogenesis and heart tissue regeneration postinjury (D'Elia et al. 2013; Barile et al. 2018). However, over‐zealous IGF signaling could promote fibrosis and/or hypertrophy (Ock et al. 2016). Thus, it will be important to understand the balance between favorable and deleterious effects of altered IGF activity. Our studies in PAPP‐A‐deficient mice indicated the benefits of moderate restraint of IGF signaling in many tissues, including visceral fat (Harrington et al. 2007; Conover et al. 2010, 2013). Further studies are necessary to determine the regulation of PAPP‐A expression in epicardial adipose tissue and its potential impact on heart function.

## Conflict of Interest

The authors report no commercial or proprietary interest in any product or concept discussed in this article.
